# Heterotopic Ossification Originating from the Lacrimal Sac of a Child: A Case Report

**DOI:** 10.18502/jovr.v20.16581

**Published:** 2025-07-30

**Authors:** Behzad Khademi, Mehdi Moallem, Mahsa Kohandel-Shirazi

**Affiliations:** ^1^Poostchi Ophthalmology Research Center, Department of Ophthalmology, School of Medicine, Shiraz University of Medical Sciences, Shiraz, Iran; ^2^Department of Pathology, School of Medicine, Shiraz University of Medical Sciences, Shiraz, Iran

**Keywords:** Hemolacria, Heterotopic Ossification, Lacrimal Sac

## Abstract

**Purpose:**

We present a case of hemolacria, which emerged as an unusual mass in the lacrimal sac.

**Case Report:**

A nine-year-old girl presented with intermittent unilateral hemolacria and episodes of dacryocystitis with no further remarkable medical or surgical history. CT scan indicated the presence of well-defined calcified tissue enclosed within the lacrimal sac. Following external dacryocystorhinostomy, well-formed firm tissue resembling bony tissue was extracted and sent for pathological evaluation, along with biopsies from the lacrimal sac. Histopathological analysis revealed heterotopic bone formation with nonspecific inflammation of the lacrimal sac. No underlying cause was discernible in the complementary assessment, and no recurrence was noted at one-year follow-up.

**Conclusion:**

A child with hemolacria was found to have heterotopic ossification in the lacrimal sac with no discernible underlying cause.

##  INTRODUCTION

Hemolacria, also known as dacryohemorrhea, is defined as the presence of blood in tears. Although uncommon, it can provoke distress and anxiety, requiring the physician to conduct a thorough diagnostic workup. The underlying cause can be found at any point along the anterior ocular surface, including the ocular adnexa, lacrimal system, and sinonasal cavities. It may indicate infectious, inflammatory, malignant, or—rarely—idiopathic sources. Obtaining a detailed history (specifically trauma and coinciding symptoms) and conducting ocular and orbital examination are required for all patients. Additionally, further evaluations, such as imaging and otolaryngologic examination, may be necessary to determine the source.

Intra-orbital bone metaplasia, also known as heterotopic ossification, is defined as the pathological formation of bony tissue within orbital soft tissues. Multiple reports of intraocular bone metaplasia have already been provided in the literature, particularly in end-stage ocular conditions such as phthisis bulbi and chronic ocular inflammation.^[1]^ These studies highlight the role of ocular trauma and inflammation as key underlying factors in the development of heterotopic ossification. On the other hand, various noninflammatory pathologies have also been reported in enucleated eyes.^[2,3]^ Extraocular orbital osseous metaplasia is also described in the context of various metastatic and dystrophic pathologies, namely hemangioma, lacrimal gland malignancy, endolymphatic malformations, and primary orbital amyloidosis.^[4]^

Here, we report a case of a nine-year-old girl presenting with hemolacria and episodes of dacryocystitis, with no specific medical history. A well-formed, hardened tissue extracted from the lacrimal sac was confirmed to be bone tissue on pathological evaluation, consistent with heterotopic ossification in the lacrimal sac.

##  CASE PRESENTATION

A nine-year-old girl was referred to the oculoplastic clinic with the chief complaint of intermittent right-sided ocular bloody discharge for the past few months. The patient had also experienced episodes of dacryocystitis. Further past medical and surgical history was unremarkable, and the patient mentioned no history of trauma. No family history of malignancy or bleeding disorder was identified. The best-corrected visual acuity was 20/20 in both eyes. Upon external eye examination, a fine, non-tender mass was palpated in the medial canthal area. No erythema, skin change, or fistula was noticed on the palpable lesion, and the punctum evaluation was unremarkable except for hemolacria. Extraocular muscle motility was normal. Intraocular pressure was within normal limits, and slit-lamp examination appeared normal with no conjunctival or vascular abnormality.

**Figure 1:**
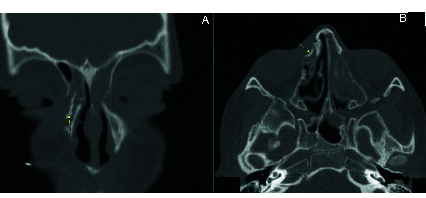
Preoperative orbital and paranasal sinuses on CT. Non-contrast-enhanced bone window CT scan, coronal (A) and axial (B) views, showing a hyperdense lesion in the lacrimal sac, with a Hounsfield scale > 1000 HU, indicating bony tissue. No soft tissue mass or orbital invasion is observed.

Computed tomography (CT) imaging of the orbital and paranasal sinuses without contrast showed a well-defined hyperdense lesion within the right lacrimal sac. The lesion was enclosed in the lacrimal sac, with no bony defects and no visible orbital or sinus invasion. Hounsfield scaling showed > 1000 HU, indicating the presence of ossification/calcification or a possible foreign body [Figure 1].

**Figure 2:**
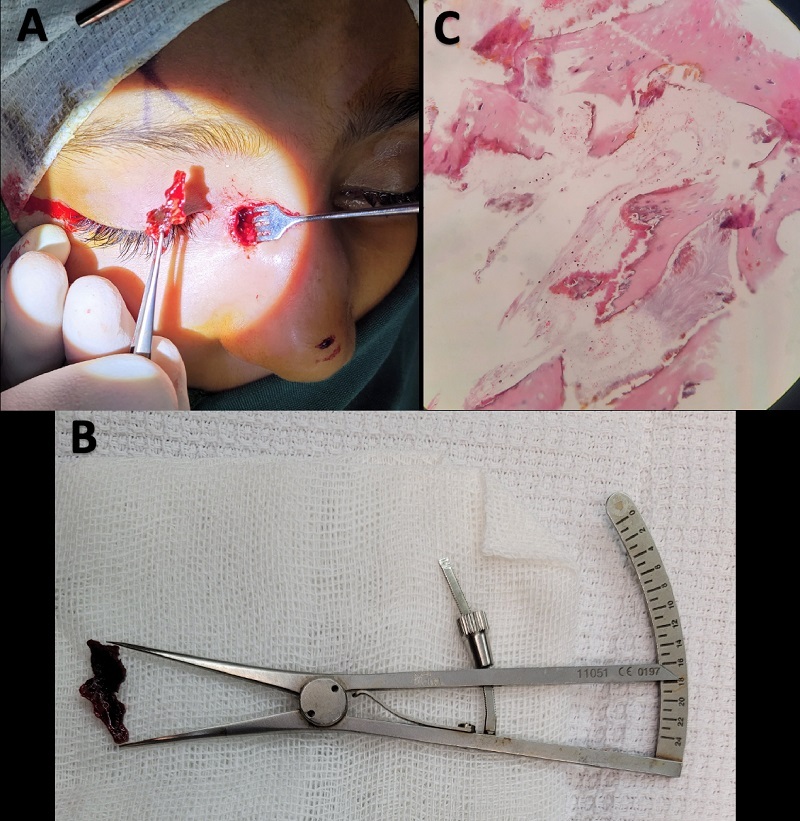
(A & B): Intraoperative view. The extracted hard-rock, well-formed bony lesion from the right lacrimal sac lumen measured 18 mm in length. (C) Histopathologic evaluation of the surgical specimen. Hematoxylin–eosin-stained specimen showing compact lamellar bone with normal osteocytes and Haversian canals, devoid of hematopoietic bone marrow elements ( × 40).

Subsequently, an external dacryocystorhinostomy (DCR) was performed under general anesthesia. Intraoperatively, the anterior lacrimal crest and the outer surface of the lacrimal sac appeared normal. Following the sac incision, an 18 mm fragment of a discrete brown, firm mass resembling bony tissue was extracted entirely from the sac lumen and sent for pathological examination [Figure 2]. Additionally, a biopsy was obtained from the clinically normal-appearing lacrimal sac wall. Considering previous episodes of dacryocystitis, it was decided to proceed with DCR to establish proper lacrimal drainage. The postoperative course was uneventful, with no recurrence of hemolacria or mass formation.

Histopathologic examination of the lesion revealed compact lamellar bone with normal osteocytes and Haversian canals, devoid of hematopoietic bone marrow elements, indicating heterotopic bone formation. There was no evidence of neoplasm or findings describing the underlying cause [Figure 2]. Histopathologic examination of the lacrimal sac wall revealed nonspecific chronic inflammation. Tissue microscopy and culture showed no growth of organisms. The evaluation of both the bony lesion and lacrimal sac biopsy and microscopy was negative for Mycobacterium species.

Systemic evaluation regarding the histopathology showed no underlying systemic disease.

##  DISCUSSION 

Here, we report a patient presenting with unilateral hemolacria, which, on CT imaging, demonstrated a bone-like tissue enclosed within the lacrimal sac. Following external DCR and removal of an 18 mm bony tissue from the lacrimal sac lumen, histopathology revealed compact lamellar bone, indicating heterotopic ossification as the probable pathology.

Hemolacria is an uncommon ophthalmic phenomenon, clinically manifested as blood-stained tears. Most cases of hemolacria are caused by trauma or conjunctival disorders. However, a thorough multidisciplinary evaluation may reveal other infectious, inflammatory, malignant, or rarely idiopathic sources. Examples include ocular surface pathologies, lacrimal gland diseases, nasolacrimal drainage system diseases, and sinonasal origin.^[5]^ Obtaining a detailed history (specifically trauma, familial bleeding tendencies, and coinciding symptoms) and performing careful ocular and orbital examination are warranted for all patients presenting with hemolacria. If indicated, otolaryngologic examination, assessment of the nasolacrimal system, and appropriate imaging can help in diagnosing the underlying condition.^[6]^ Benign or malignant lesions of the lacrimal sac are reported as potential causes of bloody tearing, as demonstrated in our case.^[7]^

Heterotopic ossification is the formation of mature and organized bone tissues in the extra-skeletal soft tissue secondary to a presumed stimulus.^[4]^ It is proposed that ectopic ossification originates from inducible osteogenic precursor cells, which could be dormant in the affected tissue or circulating in the blood. Proper stimuli, such as inflammation, trauma, or previous surgeries, could induce cytokine release, particularly bone morphogenic proteins. These proteins, in turn, stimulate the differentiation of pluripotent cells, mesenchymal cells, or fibroblasts into osteoprogenitor cells. These cells can generate osteoid, which eventually leads to calcification and the formation of mature bone.^[8]^ This pathologic course has also been reported in cases of chronic tarsus inflammation, including trachoma, syphilis, or chalazion.^[9,10]^ While bony metaplasia or calcification within the orbit has been reported in multiple cases, no study has yet reported discrete, well-formed bone within the lacrimal sac resembling our case. Lacrimal gland malignancy and vascular lesions, such as hemangioma, are considered potential causes. Stagnation of blood, followed by the population of circulating pluripotent cells, is another proposed mechanism in the development of ossified vascular lesions.^[11]^ Orbital amyloidosis is frequently reported to induce calcification in the tarsus, orbital tissues, and even the lacrimal sac and its surrounding bone.^[12]^

Lacrimal sac tumors should also be considered in the differential diagnosis. However, the sac appeared grossly normal and intact, and histopathologic evaluation of the mass and lacrimal sac wall demonstrated no evidence of malignancy. Given the patient's ethnicity and disease prevalence in Afghanistan, we also had to consider the possibility of an underlying tuberculous inflammatory reaction. However, further laboratory tests showed no such organism. Dacryolithiasis was excluded as the underlying cause, considering the presence of compact lamellar bone and Haversian canals in the pathology. Generally, inflammation is proposed as a mechanism for heterotopic ossification; however, no specific cause was identified in this case.

Although there is no reported case of malignant degeneration of heterotopic ossification, metaplasia may occur secondary to local malignancy. Hence, it is warranted to excise the suspected masses arising without other apparent stimuli.

The underlying cause in this case is subject to question. To our knowledge, this entity with such histopathologic characteristics has not previously been reported to form in the lacrimal sac. After a 12-month follow-up, the patient remained disease-free, with no recurrence of symptoms.

In summary, we presented a nine-year-old girl with unilateral hemolacria and episodes of dacryocystitis. The CT results revealed bony tissue within the lacrimal sac. During the external DCR procedure, a bony tissue was removed from the lacrimal sac lumen. This tissue was later identified as compact lamellar bone with normal osteocytes and Haversian canals, indicating heterotopic ossification upon histopathological examination. To our knowledge, there has been no previous report of discrete well-formed bone within the lacrimal sac.

##  Ethical Considerations

A consent form was obtained from the patient's guardians to publish her data and pictures without disclosing her name, and the local ethics committee approved the publication.

##  Financial Support and Sponsorship

None.

##  Conflicts of Interest

None.
